# Associations between Host Genetic Variants and Subgingival Microbiota in Patients with the Metabolic Syndrome

**DOI:** 10.3390/ijms242316649

**Published:** 2023-11-23

**Authors:** Luigi Nibali, Abish S. Stephen, Robert P. Allaker, Antonino Di Pino, Valentina Terranova, Marcella Pisano, Salvatore Di Marca, Viviana Ferrara, Roberto Scicali, Francesco Purrello, Nikolaos Donos, Matteo Regolo, Lorenzo Malatino

**Affiliations:** 1 Periodontology Unit, Centre for Host Microbiome Interactions, Faculty of Dentistry, Oral & Craniofacial Sciences, King’s College London, London WC2R 2LS, UK; luigi.nibali@kcl.ac.uk; 2 Centre for Immunobiology & Regenerative Medicine and Centre for Oral Clinical Research, Institute of Dentistry, Faculty of Medicine and Dentistry, Queen Mary University of London (QMUL), London E1 4NS, UK; a.s.stephen@qmul.ac.uk (A.S.S.); r.p.allaker@qmul.ac.uk (R.P.A.); n.donos@qmul.ac.uk (N.D.); 3 Department of Clinical and Experimental Medicine, Garibaldi-Nesima Hospital, University of Catania, 95123 Catania, Italyrobertoscicali@gmail.com (R.S.); francesco.purrello@unict.it (F.P.); 4 Department of Clinical and Experimental Medicine, Cannizzaro Hospital, University of Catania, 95123 Catania, Italymatteo.regolo.94@gmail.com (M.R.); 5 Academic Unit of Internal Medicine, Cannizzaro Hospital, Via Messina 829, 95126 Catania, Italy

**Keywords:** metabolic syndrome, periodontitis, periodontal diseases, cardiovascular diseases

## Abstract

Host genetic variants may affect oral biofilms, playing a role in the periodontitis–systemic disease axis. This is the first study to assess the associations between host genetic variants and subgingival microbiota in patients with metabolic syndrome (MetS); 103 patients with MetS underwent medical and periodontal examinations and had blood and subgingival plaque samples taken. DNA was extracted and processed, assessing a panel of selected single nucleotide polymorphisms (SNPs) first (hypothesis testing) and then expanding to a discovery phase. The subgingival plaque microbiome from these patients was profiled. Analysis of associations between host genetic and microbial factors was performed and stratified for periodontal diagnosis. Specific SNPs within *RUNX2, CAMTA1* and *VDR* genes were associated with diversity metrics with no genome-wide associations detected for periodontitis severity or Mets components at *p* < 10^−7^. Severe periodontitis was associated with pathogenic genera and species. Some SNPs correlated with specific bacterial genera as well as with microbial taxa, notably *VDR* (rs12717991) with *Streptococcus mutans* and *RUNX2* (rs3749863) with *Porphyromonas gingivalis*. In conclusion, variation in host genotypes may play a role in the dysregulated immune responses characterizing periodontitis and thus the oral microbiome, suggesting that systemic health-associated host traits further interact with oral health and the microbiome.

## 1. Introduction

“Infectogenomics” was introduced to define the effect of host genetic variants in influencing microbial colonization in a given ecological niche [1]. Applying this concept to human diseases characterized by dysbiotic biofilms, “genetic dysbiosis” implies a host genome-driven imbalance between the integrity of barrier organs and their colonizing microorganisms [2,3].

In periodontitis, inflammation is thought to drive a progressive increase in the microbial diversity, leading in turn to perturbations in the microenvironment, such as an increased availability of substrates favoring the growth of Gram-negative bacteria [4]. The resulting dysbiosis predisposes to the activation of a host response cascade, causing periodontal tissue damage and, eventually, tooth loss. Data collected over the last 20 years provided evidence about the associations between host genetic variants and the presence and counts of specific bacteria in the subgingival niche. In this regard, evidence was mainly based on the analysis of a few specific candidate host genetic variants and a few specific candidate periodontopathogenic bacteria, analyzed by checkerboard, culture or polymerase chain reaction (PCR) [5,6,7]. Only a handful of studies have employed a wider genome-wide approach [8] or a metagenomic analysis approach [9], leaving us with a rather “restricted” view of potential associations between host genetic variants and subgingival dysbiosis.

The importance of integrating host genetic data for a better understanding of subgingival dysbiosis has been recently suggested [10]. In a previous study from our group [11], biomarkers of gingival crevicular fluid were shown to be associated with Mets as well as left ventricular geometry with periodontitis. This latter finding further underscored the concept linking the burden of microinflammation on the cardiovascular system [12]. This warrants more extensive study into the host genetics and oral microbiological profile of this group of patients in order to try and add more elements to the current understanding of pathogenic factors in the periodontitis–systemic axis. Given the association between periodontitis and metabolic syndrome [13,14] and the potential effects of oral bacteria on gut microbiota [15,16,17], studying the effect of host genetic variants on the subgingival microbiota may also be particularly relevant to our understanding of connections between periodontitis and systemic health. Therefore, this study aimed to perform detailed analyses of associations between host genetic variants and subgingival microbiota in patients with Mets with or without periodontitis based on the hypothesis that host genetic variants affect the subgingival microbiota.

## 2. Results

Demographic and clinical characteristics of the 103 included subjects are reported in Table 1.

Patients were on average 58 years old, with a majority of males, and had an average BMI of nearly 32. The majority of patients (77%) were not regular dental attenders, and oral hygiene habits (tooth brushing frequency, use of interdental cleaning tools) were not up to the standard required in patients at high risk of periodontal disease. Ten patients were classified as having no–mild periodontitis, 38 were classified as having moderate periodontitis and 55 were classified as having severe periodontitis [18]. Furthermore, 38 patients were diagnosed with caries (Table 2, Supplemental Materials).

### 2.1. Microbial Diversity and Metabolic Syndrome

**Figure 1:**
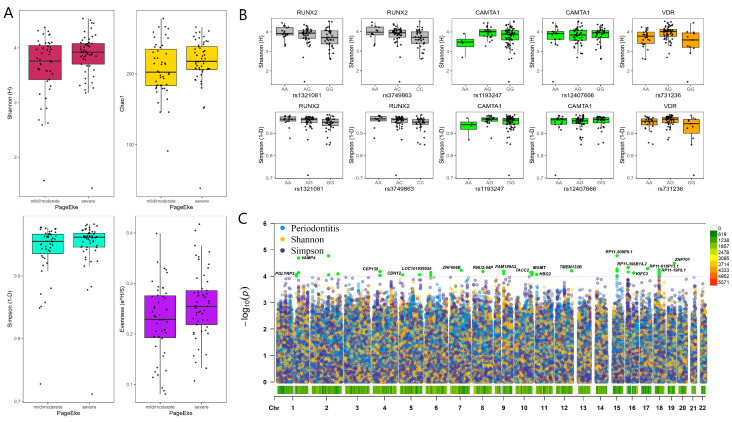
(**A**) Box and jitter plots of alpha diversity indices of the subgingival microbiota of mild–moderate and severe periodontitis MetS patients (no significant differences detected). (**B**) Box and jitter plot of alpha diversity indices that showed associations with SNPs within RUNX2, CAMTA and the VDR genes. (**C**) A Manhattan plot to compare associations between the subgingival microbiota alpha diversity measures and periodontitis severity after correcting for age, sex, smoking frequency, BMI and population structure. Suggestive associations highlighted in green.

The MED pipeline analyzed 3,439,061 sequences after quality filtering and partitioned the sequences into 1264 nodes, which mapped to 301 human microbial taxa (HMT) at ≥98.5% identity. Severe periodontitis patients had a higher alpha diversity in the subgingival plaque microbiome as measured by Shannon, Simpson, Evenness and Chao1 indices compared to no/mild or moderate periodontitis with the differences not statistically significant (Figure 1A).

Among SNPs from the selected gene panel, four SNPs within two genes were significant at *p* < 0.01 for alpha diversity: *RUNX2* (rs1321081, rs3749863) and *CAMTA1* (rs1193247, rs12407666). In addition, Evenness and Simpson indices were associated with SNPs in *TRPS1* (rs1012478)*, GLT6D1* (rs57670611)*, KCNK1* (rs701223)*, VAMP3* (rs111692854) and *VDR* (rs731236) genes at the nominal *p* < 0.05 significance (but none at the *p* < 0.01 level). The Supplemental Material (Figure S1) shows the difference between patients with different Mets components.

No genome-wide associations at *p* < 10^−7^ could be detected for alpha diversity metrics and periodontitis severity. The strongest genome-wide signals detected for periodontitis severity were at six closely associated loci within chromosome 15 downstream of the lincRNA *RP11-209E8.1* and upstream of the *WDR72* gene (*p* < 0.0001; Figure 1C). The genome-wide variants associated with Shannon diversity were in genes *CTD-3037G24.3* (rs62029200) and an SNP downstream of the lincRNA *RP11-570K4.1* (rs4714409) at *p* < 0.0001 (Figure 1C). Higher numbers of variants were associated with Simpson diversity than other alpha diversity metrics at *p* < 0.0001: *PGLYRP3*, *PGLYRP4*, *VAMP4*, *CEP135*, *CDH12*, *ZNF804B*, *RNU2-54P*, *FAM189A2*, *TACC2*, *MGMT*, *HBG2*, *TMEM132B*, *KIFC3* and *ZNF701* (Figure 1C). Although no genome-wide signals for the different components of the metabolic syndrome were detected, variants in the following genes were associated with the components of metabolic syndrome at *p* < 0.00001: (i) waist circumference: *PARD3B*, *CTD-2269F5.1*, *CSMD1*, *ADAMTSL3*; (ii) low HDL cholesterol: *PPIL6*, *SMPD2*, *MICAL1*, *BAALC*, *ESRP1*, *DPY19L4*; (iii) hypertension: *ANKS1B*; (iv) high fasting glucose: *CNNM2*, *NT5C2*, *TEX2* and lincRNA *RP3-390M24.1* (Supplemental Figure S2).

### 2.2. Genotype Associations with Microbial Taxa

Phyla distributions in patients with different periodontal diagnosis showed a predominance of Firmicutes in all three groups and phyla Fusobacteria, Spirochaetes and Synergistetes at a higher abundance in severe periodontitis compared to no–mild or moderate periodontitis (Supplemental Figure S3). No clear differences were observed when phyla distribution was studied across different genotypes.

#### 2.2.1. Genotype Associations at the Genus Level

**Figure 2:**
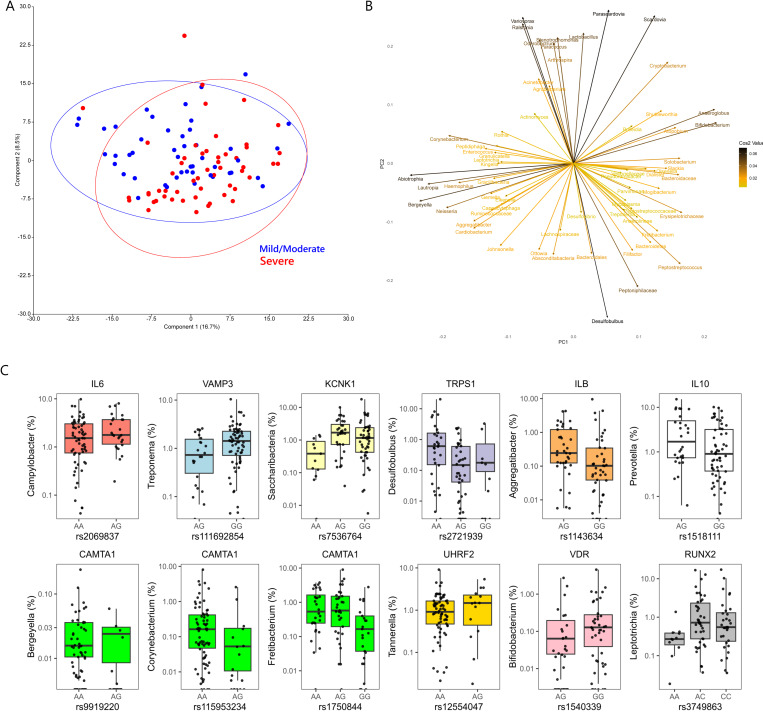
(**A**) Score plot of the first 2 principal components of CLR-transformed 16S microbial genus abundance data from the MetS periodontitis patients. (**B**) Loading plot from the principal components analysis showing microbial variables that most strongly influenced the first 2 principal components in plot A. (**C**) Box and jitter plots of SNP variants from the selected gene panel that showed significant associations with relative abundance of different subgingival genera. Please note that each SNP was filtered for all alleles present at 10% MAF for the analysis, so for SNPs below this threshold, the model was run for only two of the genotypes.

Differences were observed at the genus level between severe and mild or moderate periodontitis (Aitchison; F = 2.562; *p* = 0.0015). Principal component analysis loadings revealed that no–mild or moderate periodontitis were associated with genera such as *Actinomyces, Rothia, Corynebacterium, Granulicatella* and *Leptotrichia,* whereas severe periodontitis was associated with *Filifactor, Peptostreptococcus, Bacterioidetes, Fretibacterium, Treponema, Mogibacterium* and *Dialister* (Figure 2).

**Figure 3:**
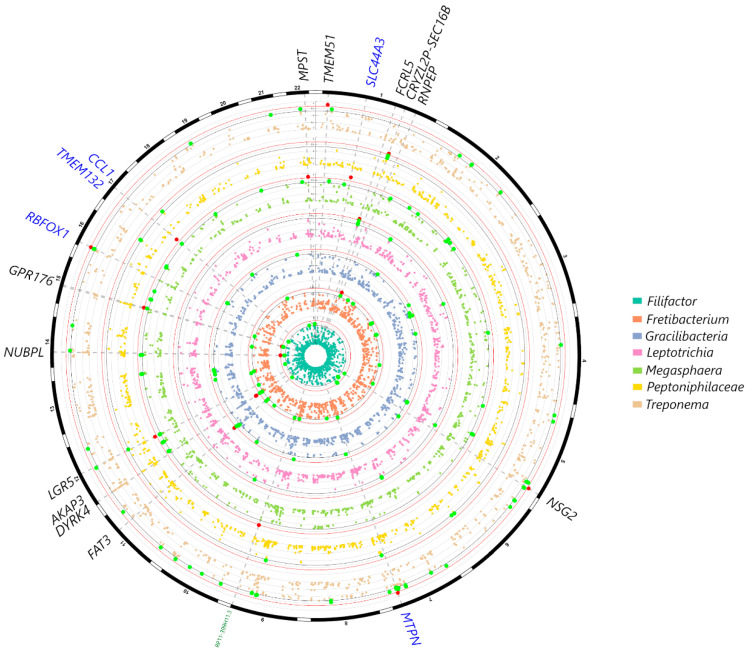
Circular Manhattan plot showing subgingival genera in the MetS patients that showed the strongest signals with genetic markers in the genome-wide analysis after correcting for age, sex, smoking frequency, BMI and population structure. Variants at *p* < 10^−3^ for at least one trait are plotted with signals at *p* < 10^−5^ highlighted in red and markers at *p* < 10^−4^ highlighted in green. Highlighted markers are annotated in the outer circle: SNPs within gene-coding regions are in black, genes adjacent to exon SNPs are in blue, non-coding RNAs and other regulatory elements are annotated in green.

From the selected gene SNP panel, *IL6* was associated with *Campylobacter*, *KCNK1* was associated with *Saccharibacteria*, *VAMP3* was associated with *Treponema,* and several variants within *CAMTA1* were associated with *Bergeyella*, *Fretibacterium*, *Actinomyces* and *Corynebacterium* at *p* < 0.001 (Figure 2). Associations were also observed with *IL1B* and *Aggregatibacter*, *TRPS1 (Desulfobulbus)*, *IL10 (Prevotella)*, *UHRF2 (Tannerella)*, *VDR (Bifidobacterium)* and *RUNX2 (Leptotrichia)* at *p* < 0.005 (Figure 2). In the genome-wide analysis, genera such as *Filifactor*, *Gracilibacteria*, *Fretibacterium*, *Leptotrichia*, *Megasphaera* and *Treponema* showed associations with several loci at *p* < 10^−5^; however, no significant associations could be detected at *p* < 10^−7^. The gene–genus pairs that showed associations at *p* < 10^−5^ included *NUBPL (Filifactor)*, *FAT3 (Gracilibacteria)*, *CAMTA1* and *AKAP3 (Fretibacterium)*, *FCRL5 (Leptotrichia)*, *MPST*, *GPR176 (Megasphaera)*, *SEC16B (Peptoniphilaceae)* and *TMEM51 (Treponema)* (Figure 3).

#### 2.2.2. Genotype Associations at the Species Level

**Figure 4:**
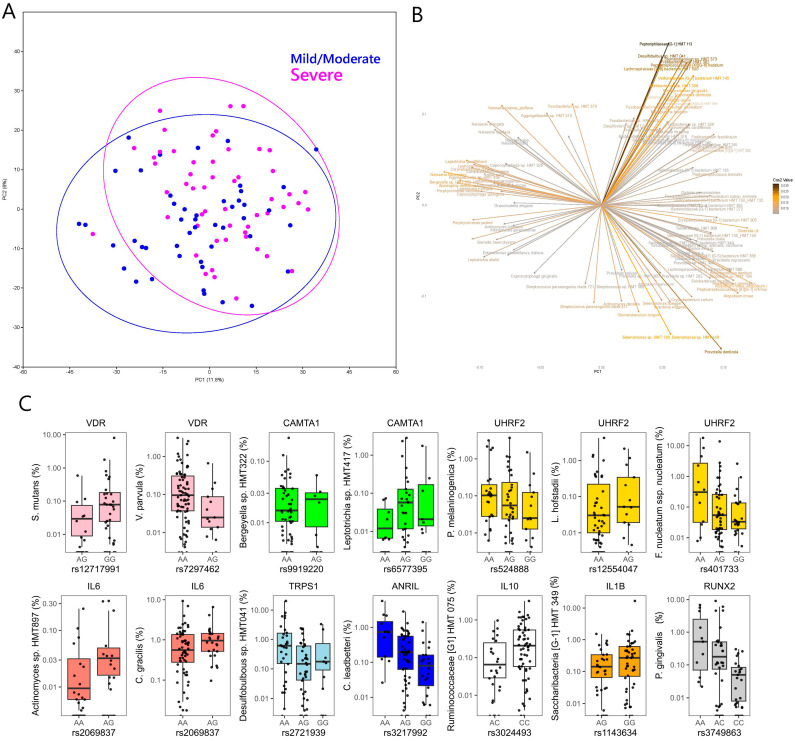
(**A**) Score plot of the first 2 principal components of CLR-transformed 16S microbial species abundance data from the MetS periodontitis patients. (**B**) Loading plot from the principal components analysis showing microbial variables that most strongly influenced the first 2 principal components in plot A. (**C**) Box and jitter plots of SNP variants from the selected gene panel that showed significant associations with relative abundance of different microbial species in the subgingival plaque. Please note that each SNP was filtered for all alleles present at 10% MAF for the analysis, so for SNPs below this threshold, the model was run for only two of the genotypes.

Consistent with the genus-level analysis, severe periodontitis was significantly different to no–mild or moderate periodontitis when the microbial taxa were considered at the species level (Aitchison; F = 2.136, *p* = 0.0015). Taxa including classical ‘red complex’ species such as *Porphyromonas gingivalis*, *Treponema denticola*, *Tannerella forsythia* and others such as *Filifactor alocis*, *Fretibacterium fastidiosum*, *Fusobacterium nucleatum* ssp. *nucleatum, Peptoniphilaceae [G-1] HMT 113*, *Lachnospiraceae [G-8] HMT 500*, *Peptostreptococcus stomatis* and *Dialister pneumosintes* were associated with severe periodontitis (Figure 4). Taxa such as *Granulicatella elegans*, *Gemella haemolysans*, *Neisseria flavescens*, *Rothia aeria*, and *Streptococcus parasanguinis* clade 411 were associated with no–mild or moderate periodontitis (Figure 4).

**Figure 5:**
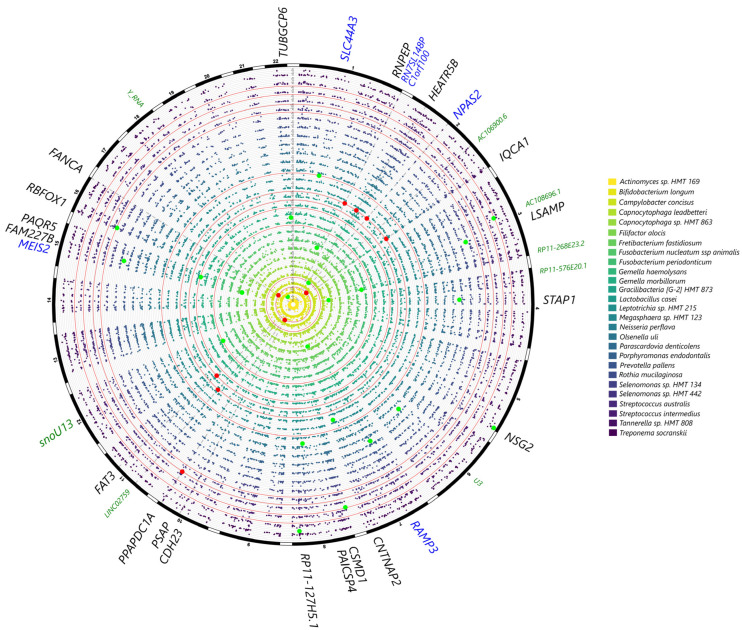
Circular Manhattan plot showing subgingival microbial species in the MetS patients that showed the strongest signals with genetic markers in the genome-wide analysis after correcting for age, sex, smoking frequency, BMI and population structure. Variants at *p* < 10^−4^ for at least one trait are plotted with signals at *p* < 10^−6^ highlighted in red and suggestive associations at *p* < 10^−5^ highlighted in green. Highlighted markers are annotated in the outer circle: SNPs within gene-coding regions are in black, genes adjacent to exon SNPs are in blue, and non-coding RNAs and other regulatory elements are annotated in green.

Among the SNPs from the selected gene panel, associations were noted at *p* < 0.0001 between *VDR* and rs12717991 and *Streptococcus mutans* (*p* < 0.0001) and at *p* < 0.001 between *CAMTA1* and *Bergeyella* sp. HMT 332 and *Leptotrichia* sp., *UHRF2 (Prevotella melaninogenica*, *Leptotrichia hofstadii*), *IL6 (Actinomyces* sp. HMT 897), and *TRPS1 (Desulfobulbus* sp. HMT 041). At *p* < 0.005, further associations were noted between *VDR* and *Veillonella parvula*, *UHRF2 (Fusobacterium nucleatum)*, *IL6* (*Campylobacter gracilis)*, *ANRIL (Capnocytophaga leadbetteri*), *IL10 (Ruminococcaceae* [G1] HMT 075), *IL1B (Saccharibacteria* TM7 [G1] HMT 349), and *RUNX2 (Porphyromonas gingivalis*) (Figure 4). Although no genome-wide associations were detected (*p* < 10^−7^), at *p* < 10^−6^, the following gene–taxa associations were detected: *Bifidobacterium longum (AC106900.6*, *CDH23*, *PSAP)*, *Gracilibacteria [G-2]* HMT 873 (*FAT3*), *Lactobacillus casei (HEATR5B, IQCA1)* and *Selenomonas* sp. HMT 442 (*PPAPDC1A*) (Figure 5).

## 3. Discussion

To the best of our knowledge, this is the first study reporting host genome-wide analysis and 16s subgingival microbiota in patients with MetS. This study broadly confirms that host genetic variants play a role in shaping the subgingival biofilm in periodontitis. Among target-studied SNPs, some showed associations with microbial diversity and with microbial species subgingivally. Measures of microbial diversity were associated with 7 of the 20 target genes. In particular, *RUNX2* (rs1321081, rs3749863) and *CAMTA1* (rs1193247, rs12407666) were associated with alpha diversity, while SNPs in *TRPS1* (rs1012478), *GLT6D1* (rs57670611), *KCNK1* (rs701223), *VAMP3* (rs111692854) and *VDR* (rs631236) genes were associated with Evenness and Simpson indices although only at the nominal *p* < 0.05 significance. A different *VDR* SNP (rs12717991) showed a strong association with *S. mutans* and a slightly weaker association with *Veillonella parvula*. For instance, *S. mutans* is notoriously a caries-associated bacterium, which appears to compete with periodontopathogenic bacteria, such as *P. gingivalis* [19]. Intriguingly, it has been shown that co-culture with *V. parvula* alters the physiology of *S. mutans*, giving it an advantage in surviving antimicrobial treatment [20,21]. These data suggest that a host genetic variant (such as this *VDR* SNP) may alter the physiology of the subgingival biofilm, potentially favoring the growth of bacteria, which in turn influences the biofilm differentiation and growth along the lines of the keystone pathogen and IMPEDE theories [22]. Findings relative to the potential effect of the VDR gene variants in the subgingival microbiota are also in agreement with an association with alpha diversity in a recent study on twins [23] and, previously, with the subgingival detection of *P. gingivalis* [24].

The associations between *IL6* SNP (rs1800795) and genera Campylobacter and *Actinomyces* sp. HMT 897 confirm its possible involvement in shaping the subgingival microbiota. This has been previously described and suspected to be mediated by an increased production of IL-6, leading to an increased inflammatory cascade, favoring in turn the growth of specific microbes [6,25]. *IL10* SNP (rs6667202) was associated with genera Prevotella and with species *Ruminococcaceae* [G1] HMT 075. This is interesting, as variants in this gene have recently emerged as potentially affecting the composition of the subgingival biofilm [3,23]. Other interesting associations emerging from this study are those between *UHRF2* and both the genera Tannerella and species *Prevotella melaninogenica*, *Leptotrichia hofstadii* and *F. nucleatum*, between TRPS1 and *Desulfobulbus*, between RUNX2 and *P. gingivalis* and between CAMTA1 and several genera and species. Some of these bacteria are well-known periodontal pathogens, while others are oral health-associated. It is hard to speculate on specific mechanisms of associations at this stage, especially for genes whose involvement in periodontal pathogenesis is still somewhat unclear. A conserved non-coding element within *CAMTA1* upstream of *VAMP3* (rs10864294) seems to emerge as a potentially important locus in the association with the subgingival microbiome, given associations with alpha diversity, as well as with specific genera and species. Interestingly, gene variants in CAMTA1/VAMP3 have been suspected to be responsible for shared predisposition to both periodontitis and cardiovascular disease [26,27], which is relevant considering the nature of the present sample (MetS).

A second part of the analysis consisted of a discovery genome-wide analysis where no statistically significant signals emerged at *p* < 10^−7^, which is consistent with other periodontal GWAS with no reported associations at this statistical level. This may in part be due to the small sample size of the present study. The closest associations with Shannon diversity data were found for CTD-3037G24.3 (rs62029200) and an SNP downstream of the lincRNA *RP11-570K4.1* (rs4714409). Although other genes showed nominal associations with subgingival genera and species, such as genera *Filifactor (*gene *NUBPL)*, *Gracilibacteria (FAT3)*, *Fretibacterium (CAMTA1*, *AKAP3)*, *Leptotrichia (FCRL5)*, *Megasphaera (MPST, GPR176)*, *Peptoniphilaceae (SEC16B)*, and *Treponema (TMEM51)*, it is difficult to speculate as to what these putative associations mean. *NUBPL* is involved in the assembly of mitochondrial Complex I, and its expression in salivary glands is reported to be associated gamma delta T cell infiltration in primary Sjogren syndrome [28]. A number of these genes, such as *FCLR5*, *FAT3*, *AKAP3*, *RUNX2*, and *CAMTA1*, are also involved in immune response pathways, so it is plausible that these variants or identified genes could be involved in a dysfunctional host microbial response in periodontitis, as the genera identified in this study to be associated with these genes was previously recognized as being associated with periodontitis. Furthermore, associations for the *CSMD1* gene were found in the species-level GWAS and waist circumference (Figure 5, Supplemental Figure S2). For instance, this gene is a regulator of the complement cascade, highlighting potential interactions between Mets and periodontitis.

The robustness of the 16s microbial analysis is confirmed by the associations detected between periodontal status and subgingival genera and species. Genera such as *Filifactor*, *Peptostreptococcus*, *Bacterioidetes*, *Fretibacterium*, *Treponema*, *Mogibacterium* and *Dialister* were increased in severe periodontitis. Furthermore, taxa including classical ‘red complex’ species such as *Porphyromonas gingivalis*, *Treponema denticola*, *Tannerella forsythia* and others with well-known associations with periodontitis, such as *Filifactor alocis*, *Fretibacterium fastidiosum*, *Fusobacterium nucleatum* ssp. *nucleatum*, *Peptoniphilaceae [G-1] HMT 113*, *Lachnospiraceae [G-8] HMT 500*, *Peptostreptococcus stomatis* and *Dialister pneumosintes*, were associated with severe periodontitis (Figure 4), while taxa such as *Granulicatella elegans*, *Gemella haemolysans*, *Neisseria flavescens*, *Rothia aeria*, and *Streptococcus parasangunis* clade 411 were associated with no–mild or moderate periodontitis. This is in line with the previous literature, as streptococci are well-known commensal bacteria, competing against periodontopathogenic bacteria, and are usually highly abundant in healthy sites [29,30]. The role of *Granulicatella* in the periodontal biofilm is less clear, while *G. adiacens* has shown the ability to co-aggregate with *F. nucleatum* [31]. Along with the classical periodontal pathogens described by Socransky et al. [32,33,34], the list of taxa associated with periodontitis in the present study includes some bacteria recently associated with periodontitis such as *Fretibacterium* [35], *Dialister pneumosintes* [36] and *Filifactor alocis* [37,38,39]. These associations are confirmatory although novel in relation to this specific population of patients with minimal dental care, and all are affected by Mets. The effect of the number of MetS components on subgingival microbiota was investigated, but only a tendency for association with microbial diversity was detected. A limitation of this analysis was the absence of diagnosis based on the 2018 classification of periodontal disease, which was not possible retrospectively in this population. The per-protocol AAP classification was instead used [18].

Some gene variants found in our periodontitis patients to link with components of Mets have recently been described as determinants in different metabolic disorders. In particular, *PARD3* has been associated with the signaling pathway related to diabetes [40]; *CSMD1* affected BMI and blood lipid levels [41]; *ADAMTSL3* was linked to metabolic impairment, especially for incipient diabetes, which was defined on the basis of both fasting and non-fasting blood glucose and the distribution of lean body mass [42,43,44]; *TEX2* was associated with diabetes and impaired lipid metabolism [45].

Previous evidence showed that periodontitis and MetS are clearly associated and possibly mediated by genetic, environmental and behavioral factors as well as by bidirectional effects of dyslipidemia, reduced glucose tolerance, oxidative stress, molecular mimicry and dysbiosis [13]. It also showed a role for host genetic variants in influencing the subgingival microbiota. This study shows that even in MetS patients, host genetic variants are likely to influence the composition of the subgingival biofilm. Among the tested SNPs (hypothesis-testing analysis), those in *RUNX2*, *CAMTA1*, *VDR, IL6* and *TRPS1* genes emerged as possibly the most likely to influence the subgingival microbiota in this patient sample, while no new SNPs clearly emerged as associated with the microbial outcomes at genome-wide analysis (hypothesis generating). This adds to our understanding of infectogenomics but calls for further studies to elucidate pathogenic pathways leading to periodontal breakdown as well as to confirm the beneficial effects of new therapeutic strategies, such as lasers, on the composition of oral microbiological niches, as recently reported by Valenti et al. [46].

The strengths of this study are the ethnic homogeneity of the included subjects, the consistency of full mouth periodontal examinations carried out by a single calibrated examiner and the novelty of combining genome-wide host data with 16s microbial analysis, which should be considered the next step for the field of infectogenomics. The main limitation of the study is the absence of controls without the metabolic syndrome (with the presence only of internal controls with MetS and no periodontitis).

Overall, some strengths should be highlighted: (1) this is the first study to report an analysis of GWAS and 16s subgingival plaque in patients with different degrees of periodontal disease, providing further evidence for infectogenomics effects on the subgingival biofilm; (2) this study suggests that systemic health-associated host traits may further interact with oral health and the microbiome.

## 4. Materials and Methods

### 4.1. Study Population

The study population has been reported before, including the finding that periodontitis may be associated with concentric left ventricular remodeling [11] and an analysis of gingival crevicular fluid [47]. The analysis described in this paper investigated associations between host genetic variants and subgingival microbiota. The STROBE checklist was followed during the conduct and reporting of the study. In brief, 103 MetS patients attending the Department of Internal Medicine (Ospedale Cannizzaro and Ospedale Garibaldi), University of Catania, for outpatient examination signed informed consent to take part in the study and were included from July 2015 to July 2017. Ethics approval was obtained by the sponsor institution, University College London (reference 4242/01), and separately by the clinical center in Catania (reference 1497/Cs, Comitato Etico Catania 1). The study was registered on clinicaltrials.gov (identifier NCT03297749).

Inclusion criteria:

- Caucasian ethnicity;
- Age 25–75;
- Diagnosis of metabolic syndrome as defined by the revised NCEP ATP III (e.g., the presence of at least 3 of the following factors) [48]:
  1. Waist circumference > 102 cm for men and >88 cm for women;
  2. High triglycerides: ≥150 mg/dL (1.7 mmol/L), or specific treatment for this lipid abnormality;
  3. Low HDL cholesterol: <40 mg/dL (1.03 mmol/L) in males, <50 mg/dL (1.29 mmol/L) in females, or specific treatment for this lipid abnormality;
  4. High blood pressure: systolic BP ≥ 130 or diastolic BP ≥ 85 mm Hg, or treatment of previously diagnosed hypertension;
  5. High fasting plasma glucose: FPG ≥ 100 mg/dL (5.6 mmol/L), or previously diagnosed type 2 diabetes.
- Presence of at least 12 teeth.

Exclusion criteria:

- Pregnancy;
- Presence of infectious diseases such as hepatitis and HIV;
- Antibiotic pre-medication required for the performance of periodontal examination;
- Previous periodontal therapy within 6 months of the study visit.

### 4.2. Medical Assessment and Sampling

As described before [11], medical and smoking histories were recorded, body mass index (BMI), waist circumference and office blood pressure were taken and blood sampling was carried out for DNA extraction. Furthermore, intima-media thickness by B-mode real-time ultrasound, pulse wave velocity and echocardiographic data were collected. Patients’ dental history was investigated, including family history of periodontal disease, frequency of dental appointments, date of last appointment and previous treatment, reasons for tooth loss and frequency and type of tooth brushing. A six sites/tooth periodontal examination was carried out by a single calibrated examiner including Full Mouth Plaque Score (FMPS) [49], Full Mouth Probing Pocket Depth (PPD), Clinical Attachment Level (CAL), Full Mouth Bleeding Score (FMBS) [49], tooth mobility and furcation involvement. Patients were classified as having periodontitis according to the criteria below [18]:

- Healthy/mild periodontitis: <2 sites on different teeth with CAL ≥ 4 mm or no sites with PPD ≥ 4 mm;
- Moderate periodontitis: ≥2 sites on different teeth with CAL ≥ 4 mm or one site with PPD ≥ 4 mm;
- Severe periodontitis: ≥2 sites on different teeth with CAL ≥ 6 mm and ≥1 site with PPD ≥ 4 mm.

Blood samples were taken by venipuncture in the antecubital vein of patients sitting in a semi-reclined position and were stored at −70° C until analysis.

Four subgingival plaque samples were taken from the disto-buccal surfaces of first molars. In the absence of these teeth, neighboring teeth were chosen (second premolars, second molars, first premolars, canines in this order). The supragingival portion of the root surface of the site was carefully cleaned, and the area was isolated from saliva before the insertion of a sterile curette to the bottom of the pocket. After a single stroke, each microbiological sample was extracted from the pocket and then pooled and placed into 1 mL of reduced transport fluid, which was then be placed in the laboratory freezer at –70 °C for storage and analysis at the end of the study.

### 4.3. Genotyping, Imputation and Genome-Wide Association Analysis

#### 4.3.1. DNA Extraction and Genotyping

Study participants were genotyped from their peripheral blood cell DNA (Nucleon BACC2 kit, Nucleon Bioscience, Coatbridge, UK) using the Illumina Infinium Global Screening Array, which measures approximately 640,000 genetic markers. The genotyping was performed at the Queen Mary University of London (QMUL) Genome Centre.

#### 4.3.2. Pre-Imputation Quality Control and Imputation

The genotype files were checked for sex assignment and filtered for >10% missing genotypes using the call rate calculations. A relatedness analysis was performed using PLINK v1.9, and the dataset was trimmed at a 0.1 relatedness cutoff in order to reduce associations due to genetic relatedness within the cohort [50]. The Sanger Imputation Server (URL: https://imputation.sanger.ac.uk/ accessed on 15th August 2023) was then used to impute the filtered and trimmed genotype files, employing EAGLE2 for phasing, Positional Burrows–Wheeler Transform (PBWT) for imputation, and the Haplotype Reference Consortium (HRC) version 1.1 as the reference panel [51,52,53]. EAGLE2 is an algorithm used for estimating haplotypes from measured genotypes in a given cohort along with PBWT utilizing the HRC reference panel to impute the missing genotypes.

#### 4.3.3. Post-Imputation Quality Control and Statistical Analysis

Genotyped and imputed SNPs underwent quality control with PLINK: multi-allelic variants, SNPs with an imputation INFO score below 0.3, genotype calls with a posterior probability < 0.9, Minor Allele Frequency (MAF) < 5%, a genotyping rate < 90%, or a deviation from the Hardy–Weinberg equilibrium (*p* < 5.7 × 10^−7^) were removed. Participants with ≥10% missing genotypes were excluded. Genome-wide association tests were performed using the mixed linear model as implemented in the R package *rMVP* [54] on the imputed SNP dataset consisting of 3,919,976 markers that passed quality control. Factors such as sex, age, BMI, smoking status and three principal components of the population structure were incorporated as covariates. Significant SNPs were annotated, and their functions were explored using SNPNexus [55].

### 4.4. Microbiome Profiling

#### 4.4.1. DNA Extraction and Sequencing

The DNA from subgingival plaque samples were extracted using the MasterPure Gram-positive DNA purification kit (Epicentre, Madison, WI, USA) with a lysozyme incubation step prior to extraction (ReadyLyse, Epicentre, Madison, WI, USA). After screening for quality, the bacterial 16S rRNA gene region V3–V4 in the samples was amplified using Nextera primers (Forward—5′-TCGTCGGCAGCGTCAGATGTGTATAAGAGACAGCCTACGGGNGGCWGCAG; Reverse—5′-GTCTCGTGGGCTCGGAGATGTGTATAAGAGACAGGACTACHVGGGTATCTAATCC). The libraries were then multiplexed, barcoded and sequenced using the Miseq V3 600 cycle kit in the 300 bp paired-end read method on the Illumina Miseq platform (Illumina, San Diego, CA, USA) at the Genome Centre, Queen Marry University of London.

#### 4.4.2. Sequence Analysis and Taxonomic Classification

The raw Illumina reads were quality filtered, trimmed, and merged with minimum overlap of 70 bases, Q30 check, a Q-score > 20 for ambiguous bases recovered in the overlapping region and up to 2 ambiguous bases allowed in the overlap [56]. The sequences were aligned using Mothur (v.1.36.1) to the Silva 16S reference alignment (release 119) [57]. Minimum entropy decomposition (MED, v2.1) was used to partition the aligned sequences into nodes at 1 nt resolution with the minimum substantive abundance parameter at 200 [58]. A BLAST search of the HOMD (v14.5) and NCBI bacterial 16S databases classified representative sequences of all MED nodes, which was used to map the nodes to the human microbial taxa (HMT) by per cent identities [59]. Nodes that mapped to multiple HMT with identical percent identities were assigned as separate taxa. The raw fastq files are deposited in the European Nucleotide Archive (Accession: PRJEB66327 & ERP151391).

#### 4.4.3. Microbial Diversity and Multivariate Analysis

Alpha diversity and multivariate statistical analyses were performed in the PAST software package (v3.22) on the HMT mapped abundance data. Interactions between periodontitis, components of Mets, and the subgingival microbiota were explored using permutational multivariate analysis of variance (PERMANOVA). Centre log ratio transformed abundance data were analyzed using principal component analysis. Dissimilarities and distances used in the testing for differences included beta diversity indices such as Bray–Curtis, Horn, Jaccard, Kulczynski and Simpson [60].

### 4.5. Statistical Analysis/Power Calculation

The sample size calculation was based on the pulse wave velocity outcome resulting in a required sample size of 103 patients [11]. Pulse wave velocity was chosen because its values are considered a surrogate marker of cardiovascular damage related to both metabolic syndrome and periodontitis. No sample size estimation was conducted for the host genome–microbial analysis. Data were entered in an MS Excel file and proofed for entry errors by personnel not directly involved in the study who investigated and resolved potential inconsistencies. The resulting database was locked and loaded in SPSS Version 23.0. Continuous, normally distributed variables are reported as means ± standard deviations (SD). Comparisons of continuous and categorical data between groups were analyzed with ANOVA and a Chi-square test, respectively. As previously reported, the dichotomous variable ‘severe periodontitis’ was given according to the criteria described above: no/mild/moderate periodontitis vs. severe periodontitis [18]. Data relative to all 3 groups (healthy–mild, moderate and severe) are also reported. Single nucleotide polymorphism (SNPs) calls from a panel of 20 available SNPs (Supplemental Table S1) were extracted from the Infinium assay raw data using Illumina GenomeStudio Software (v2.0.5). The gene panel was selected based on previous associations with periodontitis or with subgingival microbes, previous systematic reviews of genes associated with periodontitis and with subgingival microbiota complemented by GWAS and periodontal infectogenomics studies [3,6,9,61,62,63,64,65,66,67].

The initial analysis was hypothesis testing, involving the associations between the selected gene panel SNPs and microbial outcomes. Due to multiple testing, a *p* value of <0.01 was considered statistically significant for these comparisons. Alpha diversity metrics and center log ratio transformed HMT mapped abundance data at the genus, and species levels were tested against extracted loci filtered for MAF at 10%, using generalized linear models as implemented in the MASS package in R (v4.3.1) and RStudio (v2022.07.1). A discovery analysis was then carried out on imputed genetic markers, using a *p* value of 5 × 10^−7^ as the threshold for statistical significance.

## Figures and Tables

**Table 1:** Demographics and dental history of included cases. BMI= body mass index.

		**Average**
**Age**		58.12 ± 9.89
BMI		31.88 ± 4.37
		**Frequency**
Gender	Male	65 (63.1%)
	Female	48 (36.9%)
Smoking status	Non smoker	67 (65.0%)
	Current smoker	28 (27.2%)
	Former smoker	8 (7.8%)
Frequency of dental visits	Never been	2 (1.9%)
	Only in case of problems	79 (76.7%)
	1/year	12 (11.7%)
	>1/year	10 (9.7%)
Previous periodontal treatment	Yes	3 (2.9%)
	No	100 (97.1%)
Last dental visit	Never been	2 (1.9%)
	>10 years ago	7 (6.8%)
	1–10 years ago	60 (58.3%)
	<1 year ago	34 (33.0%)
Tooth-brushing frequency	<1/day	6 (5.9%)
	1/day	34 (33.0%)
	At least 2/day	63 (61.1%)
Type of toothbrush	None	1 (1.0%)
	Manual	92 (89.3%)
	Electric	10 (9.7%)
Use of interdental cleaning tools	Daily–weekly	14 (13.6%)
	Never	89 (86.4%)

**Table 2:** Dental clinical parameters in all included subjects. DMFT = Decayed Missing Filled Teeth; FMPS = Full Mouth Plaque Score; FMBS = Full Mouth Bleeding Score; PPD = Probing Pocket Depth; CAL = Clinical Attachment Level.

	Average	Number
Number of teeth (excluding third molars)	22.77 ± 4.19	2345
DMFT	12.58 ± 6.07	-
Decayed teeth	0.63 ± 0.96	65
Patients with caries detected	-	38 (36.89%)
FMPS	72.11 ± 22.51	-
FMBS	23.92 ± 19.62	-
Average PPD	2.44 ± 0.73	-
Average CAL	3.05 ± 1.12	-
% PPDs 1–4 mm	93.17 ± 0.88%	-
% PPDs 5–6 mm	5.92 ± 0.75%	-
% of PPDs > 6 mm	0.94 ± 0.23%	-
No–Mild periodontitis	-	10 (9.7%)
Moderate periodontitis	-	38 (36.9%)
Severe periodontitis	-	55 (53.3%)

## Data Availability

The data presented in this study are available on request from the corresponding author.

## Supplementary Materials

The following supporting information can be downloaded at https://www.mdpi.com/article/10.3390/ijms242316649/s1.

## References

[1] Kellam: P., Weiss R.A. (2006). Infectogenomics: Insights from the Host Genome into Infectious Diseases. Cell.

[2] Nibali L., Henderson B., Sadiq S.T., Donos N. (2014). Genetic dysbiosis: The role of microbial insults in chronic inflammatory diseases. J. Oral Microbiol..

[3] Zoheir N., Kurushima Y., Lin G.-H., Nibali L. (2022). Periodontal infectogenomics: A systematic review update of associations between host genetic variants and subgingival microbial detection. Clin. Oral Investig..

[4] Hajishengallis G., Lamont R.J. (2012). Beyond the red complex and into more complexity: The polymicrobial synergy and dysbiosis (PSD) model of periodontal disease etiology. Mol. Oral Microbiol..

[5] Socransky S.S., Haffajee A.D. (2005). Periodontal microbial ecology. Periodontology 2000.

[6] Nibali L., Ready D., Parkar M., Brett P., Wilson M., Tonetti M., Griffiths G. (2007). Gene Polymorphisms and the Prevalence of Key Periodontal Pathogens. J. Dent. Res..

[7] Nibali L., Donos N., Brett P.M., Parkar M., Ellinas T., Llorente M., Griffiths G.S. (2008). A familial analysis of aggressive periodontitis—Clinical and genetic findings. J. Periodontal Res..

[8] Divaris K., Monda K.L., North K.E., Olshan A.F., Reynolds L.M., Hsueh W.-C., Lange E.M., Moss K., Barros S.P., Weyant R.J. (2013). Exploring the genetic basis of chronic periodontitis: A genome-wide association study. Hum. Mol. Genet..

[9] Ye Y., Carlsson G., Wondimu B., Fahlén A., Karlsson-Sjöberg J., Andersson M., Engstrand L., Yucel-Lindberg T., Modéer T., Pütsep K. (2011). Mutations in the ELANE Gene are Associated with Development of Periodontitis in Patients with Severe Congenital Neutropenia. J. Clin. Immunol..

[10] Zhang S., Yu N., Arce R.M. (2019). Periodontal inflammation: Integrating genes and dysbiosis. Periodontol. 2000.

[11] Nibali L., Donos N., Terranova V., Di Pino A., Di Marca S., Ferrara V., Pisano M., Scicali R., Rabuazzo A.M., Purrello F. (2018). Left ventricular geometry and periodontitis in patients with the metabolic syndrome. Clin. Oral Investig..

[12] Zanoli L., Ozturk K., Cappello M., Inserra G., Geraci G., Tuttolomondo A., Torres D., Pinto A., Duminuco A., Riguccio G. (2019). Inflammation and Aortic Pulse Wave Velocity: A Multicenter Longitudinal Study in Patients With Inflammatory Bowel Disease. J. Am. Heart Assoc..

[13] Nibali L., Tatarakis N., Needleman I., Tu Y.-K., D’Aiuto F., Rizzo M., Donos N. (2013). Association Between Metabolic Syndrome and Periodontitis: A Systematic Review and Meta-analysis. J. Clin. Endocrinol. Metab..

[14] Suwanprasit W., Lertpimonchai A., Thienpramuk L., Vathesatogkit P., Sritara P., Tamsailom S. (2021). Metabolic syndrome and severe periodontitis were associated in Thai adults: A cross-sectional study. J. Periodontol..

[15] Lourenςo T.G.B., Spencer S.J., Alm E.J., Colombo A.P.V. (2018). Defining the gut microbiota in individuals with periodontal diseases: An exploratory study. J. Oral Microbiol..

[16] Olsen I., Yamazaki K. (2019). Can oral bacteria affect the microbiome of the gut?. J. Oral Microbiol..

[17] Bao J., Li L., Zhang Y., Wang M., Chen F., Ge S., Chen B., Yan F. (2022). Periodontitis may induce gut microbiota dysbiosis via salivary microbiota. Int. J. Oral Sci..

[18] Page R.C., Eke P.I. (2007). Case Definitions for Use in Population-Based Surveillance of Periodontitis. J. Periodontol..

[19] Tu Y., Ling X., Chen Y., Wang Y., Zhou N., Chen H. (2017). Effect of S. Mutans and S. Sanguinis on Growth and Adhesion of P. Gingivalis and Their Ability to Adhere to Different Dental Materials. Experiment.

[20] Luppens S.B.I., Kara D., Bandounas L., Jonker M.J., Wittink F.R.A., Bruning O., Breit T.M., Cate J.M.T., Crielaard W. (2008). Effect of *Veillonella parvula* on the antimicrobial resistance and gene expression of *Streptococcus mutans* grown in a dual-species biofilm. Oral Microbiol. Immunol..

[21] Liu S., Chen M., Wang Y., Zhou X., Peng X., Ren B., Li M., Cheng L. (2020). Effect of Veillonella parvula on the physiological activity of Streptococcus mutans. Arch. Oral Biol..

[22] Van Dyke T.E., Bartold P.M., Reynolds E.C. (2020). The Nexus Between Periodontal Inflammation and Dysbiosis. Front. Immunol..

[23] Kurushima Y., Wells P., Bowyer R., Zoheir N., Doran S., Richardson J., Sprockett D., Relman D., Steves C., Nibali L. (2022). Host Genotype Links to Salivary and Gut Microbiota by Periodontal Status. J. Dent. Res..

[24] Torrungruang K., Chantarangsu S., Sura T., Thienpramuk L. (2020). Interplay between vitamin D receptor *Fok*I polymorphism and smoking influences *Porphyromonas gingivalis* proportions in subgingival plaque. J. Clin. Periodontol..

[25] Nibali L., Madden I., Chillida F.F., Heitz-Mayfield L., Brett P., Donos N. (2010). *IL6 −*174 Genotype Associated with *Aggregatibacter actinomycetemcomitans* in Indians. Oral Dis..

[26] Schaefer A.S., Bochenek G., Jochens A., Ellinghaus D., Dommisch H., Güzeldemir-Akçakanat E., Graetz C., Harks I., Jockel-Schneider Y., Weinspach K. (2015). Genetic Evidence for *PLASMINOGEN* as a Shared Genetic Risk Factor of Coronary Artery Disease and Periodontitis. Circ. Cardiovasc. Genet..

[27] Aarabi G., Zeller T., Seedorf H., Reissmann D., Heydecke G., Schaefer A., Seedorf U. (2017). Genetic Susceptibility Contributing to Periodontal and Cardiovascular Disease. J. Dent. Res..

[28] Li N., Li Y., Hu J., Wu Y., Yang J., Fan H., Li L., Luo D., Ye Y., Gao Y. (2022). A Link Between Mitochondrial Dysfunction and the Immune Microenvironment of Salivary Glands in Primary Sjogren’s Syndrome. Front. Immunol..

[29] Xie H., Hong J., Sharma A., Wang B. (2012). *Streptococcus cristatus* ArcA interferes with *Porphyromonas gingivalis* pathogenicity in mice. J. Periodontal Res..

[30] Ho M.-H., Lamont R.J., Xie H. (2017). Identification of Streptococcus cristatus peptides that repress expression of virulence genes in Porphyromonas gingivalis. Sci. Rep..

[31] Karched M., Bhardwaj R.G., Asikainen S.E. (2015). Coaggregation and biofilm growth of Granulicatella spp. with Fusobacterium nucleatum and Aggregatibacter actinomycetemcomitans. BMC Microbiol..

[32] Haffajee A.D., Socransky S.S., Smith C., Dibart S. (1991). Relation of baseline microbial parameters to future periodontal attachment loss. J. Clin. Periodontol..

[33] Haffajee A.D., Socransky S.S., Dibart S., Kent R.L. (1996). Response to periodontal therapy in patients with high or low levels of P. gingivalis, P. intermedia, R nigrescens and B. forsythus. J. Clin. Periodontol..

[34] Socransky S.S., Haffajee A.D., Cugini M.A., Smith C., Kent R.L. (1998). Microbial complexes in subgingival plaque. J. Clin. Periodontol..

[35] Oliveira R., Fermiano D., Feres M., Figueiredo L., Teles F., Soares G., Faveri M. (2016). Levels of Candidate Periodontal Pathogens in Subgingival Biofilm. J. Dent. Res..

[36] Ghayoumi N., Chen C., Slots J. (2002). *Dialister pneumosintes*, a new putative jreiodontal pathogen. J. Periodontal Res..

[37] Aruni A.W., Mishra A., Dou Y., Chioma O., Hamilton B.N., Fletcher H.M. (2015). Filifactor alocis—A new emerging periodontal pathogen. Microbes Infect..

[38] Ozuna H., Snider I., Belibasakis G.N., Oscarsson J., Johansson A., Uriarte S.M. (2022). Aggregatibacter actinomycetemcomitans and Filifactor alocis: Two exotoxin-producing oral pathogens. Front. Oral Health.

[39] Razooqi Z., Åberg C.H., Kwamin F., Claesson R., Haubek D., Oscarsson J., Johansson A. (2022). *Aggregatibacter actinomycetemcomitans* and *Filifactor alocis* as Associated with Periodontal Attachment Loss in a Cohort of Ghanaian Adolescents. Microorganisms.

[40] Peña-Chilet M., Esteban-Medina M., Falco M.M., Rian K., Hidalgo M.R., Loucera C., Dopazo J. (2019). Using mechanistic models for the clinical interpretation of complex genomic variation. Sci. Rep..

[41] Ke J., Gao W., Wang B., Cao W., Lv J., Yu C., Huang T., Sun D., Liao C., Pang Y. (2023). Exploring the Genetic Association between Obesity and Serum Lipid Levels Using Bivariate Methods. Twin Res. Hum. Genet..

[42] de Vries P.S., van Herpt T.T.W., Ligthart S., Hofman A., Ikram M.A., van Hoek M., Sijbrands E.J.G., Franco O.H., de Maat M.P.M., Leebeek F.W.G. (2016). ADAMTS13 activity as a novel risk factor for incident type 2 diabetes mellitus: A population-based cohort study. Diabetologia.

[43] Zillikens M.C., Demissie S., Hsu Y.-H., Yerges-Armstrong L.M., Chou W.-C., Stolk L., Livshits G., Broer L., Johnson T., Koller D.L. (2017). Large meta-analysis of genome-wide association studies identifies five loci for lean body mass. Nat. Commun..

[44] Zhu Z., Guo Y., Shi H., Liu C.-L., Panganiban R.A., Chung W., O’Connor L.J., Himes B.E., Gazal S., Hasegawa K. (2019). Shared genetic and experimental links between obesity-related traits and asthma subtypes in UK Biobank. J. Allergy Clin. Immunol..

[45] Baca P., Barajas-Olmos F., Mirzaeicheshmeh E., Zerrweck C., Guilbert L., Sánchez E.C., Flores-Huacuja M., Villafán R., Martínez-Hernández A., García-Ortiz H. (2022). DNA methylation and gene expression analysis in adipose tissue to identify new loci associated with T2D development in obesity. Nutr. Diabetes.

[46] Valenti C., Pagano S., Bozza S., Ciurnella E., Lomurno G., Capobianco B., Coniglio M., Cianetti S., Marinucci L. (2021). Use of the Er:YAG Laser in Conservative Dentistry: Evaluation of the Microbial Population in Carious Lesions. Materials.

[47] Nibali L., Stephen A., Hagi-Pavli E., Allaker R., Di Pino A., Terranova V., Pisano M., Di Marca S., Ferrara V., Scicali R. (2022). Analysis of gingival crevicular fluid biomarkers in patients with metabolic syndrome. J. Dent..

[48] Grundy S.M., Brewer H.B., Cleeman J.I., Smith S.C., Lenfant C. (2004). Definition of Metabolic Syndrome: Report of the National Heart, Lung, and Blood Institute/American Heart Association conference on scientific issues related to definition. Circulation.

[49] Guerrero A., Griffiths G.S., Nibali L., Suvan J., Moles D.R., Laurell L., Tonetti M.S. (2005). Adjunctive benefits of systemic amoxicillin and metronidazole in non-surgical treatment of generalized aggressive periodontitis: A randomized placebo-controlled clinical trial. J. Clin. Periodontol..

[50] Chang C.C., Chow C.C., Tellier L.C., Vattikuti S., Purcell S.M., Lee J.J. (2015). Second-generation PLINK: Rising to the challenge of larger and richer datasets. GigaScience.

[51] Loh P.-R., Danecek P., Palamara P.F., Fuchsberger C., Reshef Y.A., Finucane H.K., Schoenherr S., Forer L., McCarthy S., Abecasis C.F.G.R. (2016). Reference-based phasing using the Haplotype Reference Consortium panel. Nat. Genet..

[52] Durbin R. (2014). Efficient haplotype matching and storage using the positional Burrows–Wheeler transform (PBWT). Bioinformatics.

[53] McCarthy S., Das S., Kretzschmar W., Delaneau O., Wood A.R., Teumer A., Kang H.M., Fuchsberger C., Danecek P., Sharp K. (2016). A reference panel of 64,976 haplotypes for genotype imputation. Nat. Genet..

[54] Yin L., Zhang H., Tang Z., Xu J., Yin D., Zhang Z., Yuan X., Zhu M., Zhao S., Li X. (2021). rMVP: A Memory-efficient, Visualization-enhanced, and Parallel-accelerated Tool for Genome-wide Association Study. Genom. Proteom. Bioinform..

[55] Oscanoa J., Sivapalan L., Gadaleta E., Ullah A.Z.D., Lemoine N.R., Chelala C. (2020). SNPnexus: A web server for functional annotation of human genome sequence variation (2020 update). Nucleic Acids Res..

[56] Eren A.M., Vineis J.H., Morrison H.G., Sogin M.L. (2013). A Filtering Method to Generate High Quality Short Reads Using Illumina Paired-End Technology. PLoS ONE.

[57] Schloss P.D., Westcott S.L., Ryabin T., Hall J.R., Hartmann M., Hollister E.B., Lesniewski R.A., Oakley B.B., Parks D.H., Robinson C.J. (2009). Introducing mothur: Open-Source, Platform-Independent, Community-Supported Software for Describing and Comparing Microbial Communities. Appl. Environ. Microbiol..

[58] Eren A.M., Morrison H.G., Lescault P.J., Reveillaud J., Vineis J.H., Sogin M.L. (2014). Minimum entropy decomposition: Unsupervised oligotyping for sensitive partitioning of high-throughput marker gene sequences. ISME J..

[59] Chen T., Yu W.-H., Izard J., Baranova O.V., Lakshmanan A., Dewhirst F.E. (2010). The Human Oral Microbiome Database: A web accessible resource for investigating oral microbe taxonomic and genomic information. Database.

[60] Hammer Ø., Harper D.A., Ryan P.D. (2001). Past: Paleontological statistics software package for educaton and data anlysis. Palaeontol. Electron..

[61] Cavalla F., Biguetti C.C., Melchiades J.L., Tabanez A.P., Azevedo M.D.C.S., Trombone A.P.F., Faveri M., Feres M., Garlet G.P. (2018). Genetic Association with Subgingival Bacterial Colonization in Chronic Periodontitis. Genes.

[62] Rhodin K., Divaris K., North K., Barros S., Moss K., Beck J., Offenbacher S. (2014). Chronic Periodontitis Genome-wide Association Studies. J. Dent. Res..

[63] Shusterman A., Durrant C., Mott R., Polak D., Schaefer A., Weiss E., Iraqi F., Houri-Haddad Y. (2013). Host Susceptibility to Periodontitis. J. Dent. Res..

[64] Shusterman A., Munz M., Richter G., Jepsen S., Lieb W., Krone B., Hoffman P., Laudes M., Wellmann J., Berger K. (2017). The *PF4/PPBP/CXCL5* Gene Cluster Is Associated with Periodontitis. J. Dent. Res..

[65] Munz M., Richter G.M., Loos B.G., Jepsen S., Divaris K., Offenbacher S., Teumer A., Holtfreter B., Kocher T., Bruckmann C. (2018). Meta-analysis of genome-wide association studies of aggressive and chronic periodontitis identifies two novel risk loci. Eur. J. Hum. Genet..

[66] Munz M., Willenborg C., Richter G.M., Jockel-Schneider Y., Graetz C., Staufenbiel I., Wellmann J., Berger K., Krone B., Hoffmann P. (2017). A genome-wide association study identifies nucleotide variants at SIGLEC5 and DEFA1A3 as risk loci for periodontitis. Hum. Mol. Genet..

[67] Divaris K., Monda K., North K., Olshan A., Lange E., Moss K., Barros S., Beck J., Offenbacher S. (2012). Genome-wide Association Study of Periodontal Pathogen Colonization. J. Dent. Res..

