# Supplementary material for: Associations between Host Genetic Variants and Subgingival Microbiota in Patients with the Metabolic Syndrome
Source: Int J Mol Sci. 2023 Nov 23;24(23):16649. doi: 10.3390/ijms242316649 (PMC10706808; doi:10.3390/ijms242316649)
Supplement: Supplementary file 1 [file ijms-24-16649-s001.zip › ijms-2667713-supplementary.pdf]

| Gene | Start | End | Chr |
|---|---|---|---|
| VAMP3 | 7831356 | 7841492 | 1 |
| GLT6D1 | 138515494 | 138531386 | 9 |
| IL10 | 206940947 | 206945839 | 1 |
| IL1B | 113587328 | 113594356 | 2 |
| IL6 | 22766819 | 22771617 | 7 |
| ANRIL (CDKN2B) | 22002902 | 22009312 | 9 |
| NPY | 24323809 | 24331481 | 7 |
| NCR2 | 41303346 | 41318627 | 6 |
| CAMTA1 | 6845514 | 7829766 | 1 |
| PLG | 161123225 | 161175129 | 6 |
| PKN2 | 89149956 | 89301938 | 1 |
| KCNK1 | 233749852 | 233808260 | 1 |
| RUNX2 | 45296067 | 45518819 | 6 |
| TRPS1 | 116420724 | 116681202 | 8 |
| FBXO38 | 147763521 | 147822399 | 5 |
| UHRF2 | 6413199 | 6507054 | 9 |
| SIGLEC5 | 52114213 | 52133654 | 19 |
| MTND1P5 | 104097895 | 104098821 | 8 |
| VDR | 48235320 | 48298777 | 12 |
| Fc-gammaRIIA | 161475247 | 161489619 | 1 |

Supplement Table S1. Table listing gene panel and their corresponding HG19 co-ordinates used to extract SNPs from the genotyping raw data.

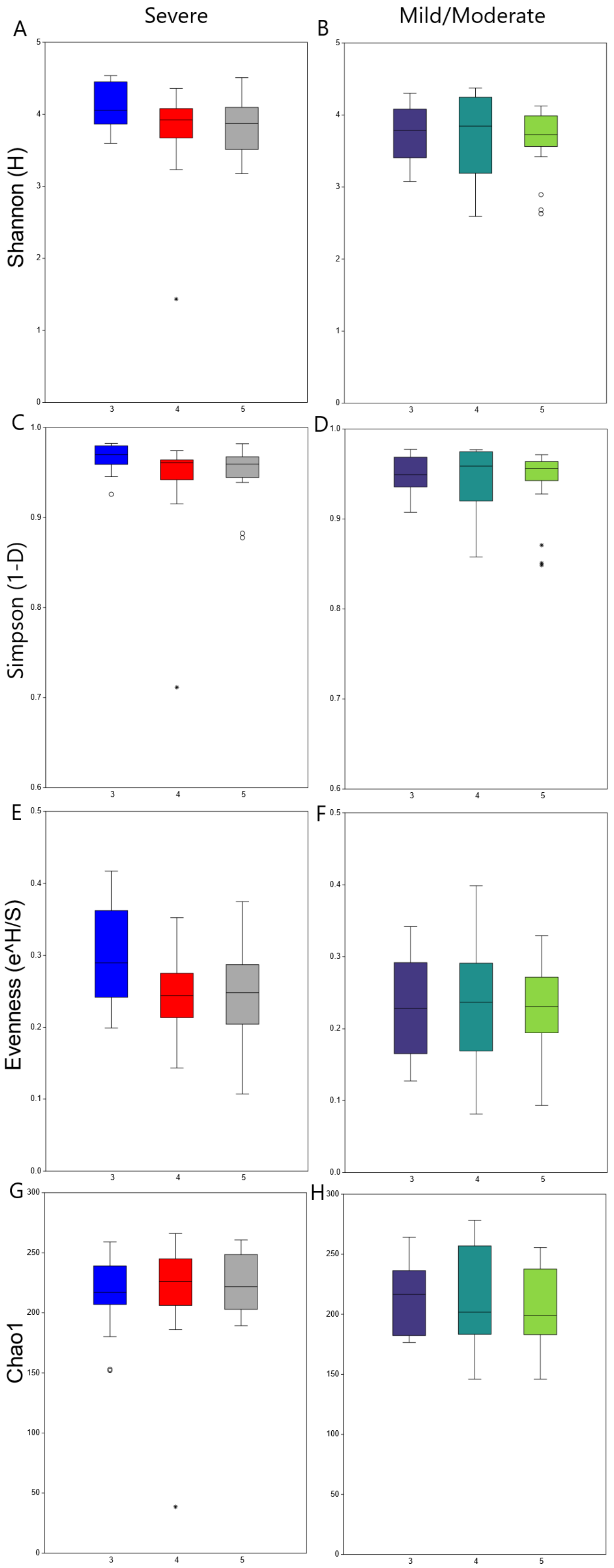


Supplement Figure S1. Box plots showing different alpha diversity metrics for patients that had 3,4 or 5 metabolic syndrome components. Majority of patients with 3 components had normal triglycerides and cholesterol levels.

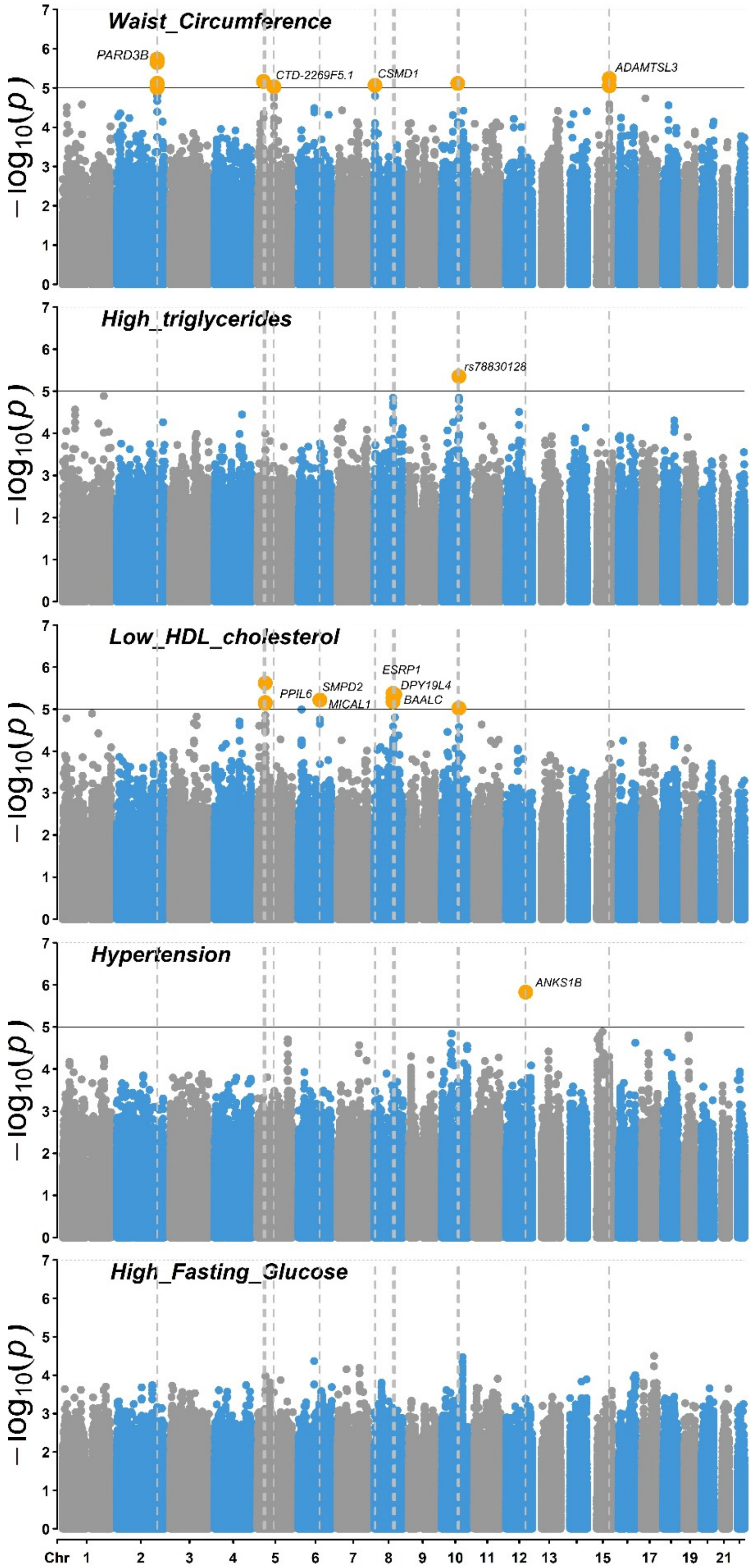


Supplement Figure S2. Manhattan plots of gene variants against each component of the metabolic syndrome in the genome-wide analysis, after correcting for age, sex, smoking frequency, BMI and population structure. Variants at $p<10^{-5}$ are annotated and highlighted in orange.

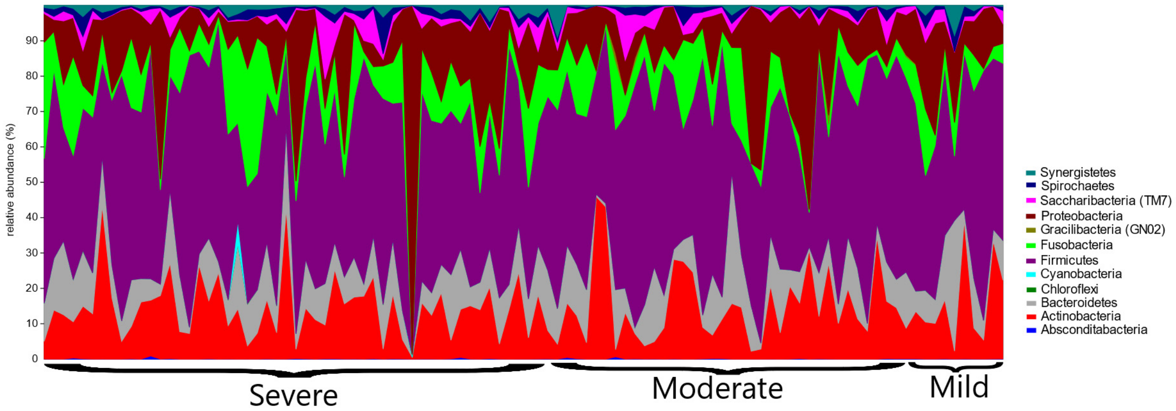


Supplement  Figure S3. Stacked chart of the observed phyla distributions for all patients in this study, with the periodontitis diagnosis indicated in the x-axis.
